# Prevalence and Genetic Diversity of Bat Hepatitis B Viruses in Bat Species Living in Gabon

**DOI:** 10.3390/v16071015

**Published:** 2024-06-25

**Authors:** Danielle S. Koumba Mavoungou, Linda Bohou Kombila, Neil M. Longo Pendy, Schedy E. Koumba Moukouama, Sonia Etenna Lekana-Douki, Gaël D. Maganga, Eric M. Leroy, Avelin F. Aghokeng, Nadine N’dilimabaka

**Affiliations:** 1 Unité Emergence des Maladies Virales, Département de Virologie, Centre Interdisciplinaire de Recherches Médicales de Franceville (CIRMF), Franceville BP 769, Gabon; danstyvie@gmail.com (D.S.K.M.); bohoukombilalinda@gmail.com (L.B.K.); koumbaschedy@gmail.com (S.E.K.M.); s_lekana@yahoo.fr (S.E.L.-D.); gael_maganga@yahoo.fr (G.D.M.); 2 Unité Ecologie des Systèmes Vectoriels, Département de parasitologie, Centre Interdisciplinaire de Recherches Médicales de Franceville (CIRMF), Franceville BP 769, Gabon; longo2michel@gmail.com; 3 Institut National Supérieur d’Agronomie et de Biotechnologies (INSAB), Université des Sciences et Techniques de Masuku (USTM), Franceville BP 941, Gabon; 4 Institut de Recherche pour le Développement (IRD), Maladies Infectieuses et Vecteurs, Écologie, Génétique, Évolution et Contrôle (MIVEGEC), (Université de Montpellier-IRD 224-CNRS5290), 34394 Montpellier, France; eric.leroy@ird.fr (E.M.L.); avelin.aghokeng@ird.fr (A.F.A.); 5 Département de Biologie, Faculté des Sciences, Université des Sciences et Techniques de Masuku (USTM), Franceville BP 901, Gabon

**Keywords:** hepatitis B virus, genetic diversity, bat species, interspecies transmission, Gabon

## Abstract

Hepatitis B virus (HBV) infection leads to around 800,000 deaths yearly and is considered to be a major public health problem worldwide. However, HBV origins remain poorly understood. Here, we looked for bat HBV (BtHBV) in different bat species in Gabon to investigate the role of these animals as carriers of ancestral hepadnaviruses because these viruses are much more diverse in bats than in other host species. DNA was extracted from 859 bat livers belonging to 11 species collected in caves and villages in the southeast of Gabon and analyzed using PCRs targeting the surface gene. Positive samples were sequenced using the Sanger method. BtHBV DNA was detected in 64 (7.4%) individuals belonging to eight species mainly collected in caves. Thirty-six (36) sequences among the 37 obtained after sequencing were phylogenetically close to the RBHBV strain recently isolated in Gabonese bats, while the remaining sequence was close to a rodent HBV strain isolated in America. The generalized linear mixed model showed that the variable species best explained the occurrence of BtHBV infection in bats. The discovery of a BtHBV strain homologous to a rodent strain in bats raises the possibility that these animals may be carriers of ancestral hepadnaviruses.

## 1. Introduction

Hepatitis B virus (HBV) infection is a major public health problem worldwide. Indeed, about 2 billion people worldwide are carriers of past or present serological markers of this infection. HBV can cause chronic infections that can lead to liver cirrhosis and hepatocellular carcinoma, which are responsible for more than 800,000 deaths every year [1,2]. Prevalences of HBV infection differ by geographic area and are mostly underestimated. Sub-Saharan Africa and the Western Pacific are the areas of highest endemicity [1]. HBV is a circular partially double-stranded DNA virus belonging to the *Orthohepadnavirus* genus. It is the prototype virus of the *Hepadnaviridae* family that includes two genera as follows: *Orthohepadnavirus* and *Avihepadnavirus*. HBV origins remain poorly understood. However, phylogenetic analyses suggest that humans have had HBV for several thousand years [3,4].

*Orthohepadnavirus* infects a wide range of mammals including humans. Among these animals are rodents like marmots (*Marmota monax*), ground squirrels (*Spermophilus beecheyi*), and arctic ground squirrels (*Spermophilus parryii*) in America [5,6,7]. However, these rodent strains have never been found in other animal species, suggesting a strong host specificity. Non-human primates such as chimpanzees, gorillas, gibbons, and orangutans can also carry primate HBV. However, until now, there is no evidence that these animals’ viral strains can infect human. Nevertheless, a strain found in the woolly monkey is close to the human strain, suggesting both a possible human infection through this strain and a possible zoonotic origin of human HBV [8,9].

In addition, in the last decade, orthohepadnaviruses able to infect human cells have been found in bats. Orthohepadnaviruses are much more diverse in bats than in other host species [10,11], suggesting that these animals could be the host ancestors of orthohepadnaviruses. Indeed, a single bat species can harbor various strains of these viruses and a single species of *Orthohepadnavirus* can infect different bat species. Overall, four strains of bat hepatitis B virus (BtHBV) have been described in bats, including long-fingered bat HBV (LBHBV) detected in Myanmar, tent-making bat HBV (TBHBV) detected in Panama, roundleaf bat HBV (RBHBV), and horseshoe bat HBV (HBHBV) detected in Gabon [10,11]. It has been shown that TBHBV found in Panama can infect primary human hepatocytes through binding to the same receptor as human HBV, suggesting that bat *Orthohepadnavirus* are ancestors of primate HBV and underscoring the zoonotic potential of some hepadnaviruses [11].

Bats (order *Chiroptera*) represent about 25% of mammals [12]. They have been described as potential reservoirs of many zoonotic pathogens recently identified [13] with high human life-threatening potential, such as Ebola virus [14], Marburg virus [15], Hendra virus [16], Nipah virus [17], MERS-CoV [18], SARS-CoV [19], and the recently discovered SARS-CoV-2 [20]. In addition, bats can also carry many other non-zoonotic viruses like bat coronaviruses (CoVs), bat astroviruses, herpesviruses, and adenoviruses [21]. This ability to host many pathogens could be due to their capacity to facilitate viral persistence and chronic infection, making these animals suitable hosts for many pathogens. Because of their proximity to humans, they represent a permanent threat to human health [12]. Considering that bats carry the highest zoonotic viruses by species out of all animals and that some orthohepadnaviruses found in bats can infect human hepatocytes, we investigated BtHBV in bats and looked for the determinants that might promote BtHBV infection in these animals.

## 2. Materials and Methods

### 2.1. Study Area and Sample Origins

A retrospective study was conducted using bat liver samples that were collected from various studies focused on the research of filoviruses (Ebola virus and Marburg virus), flaviviruses, phleboviruses, paramyxoviruses (unpublish data), arenaviruses [22], and coronaviruses [23]; it was archived in the biorepository of the Centre Interdisciplinaire de Recherches Médicales de Franceville (CIRMF). The bat samples were collected across three provinces in the eastern region of Gabon as follows: Haut-Ogooué, Ogooué-Ivindo, and Ogooué-Lolo. Bats were captured monthly in March–April 2013 (Djibilong cave), July 2013 (Faucon, zadié and Batouala caves), February 2014 (Ngoungourouma cave), April–May 2017 (Loa loa and Bombenda villages), and June–July 2019 (Ekata, Grand Etoumbi, Ilahounene, Imbong and Mendemba villages).

**Figure 1:**
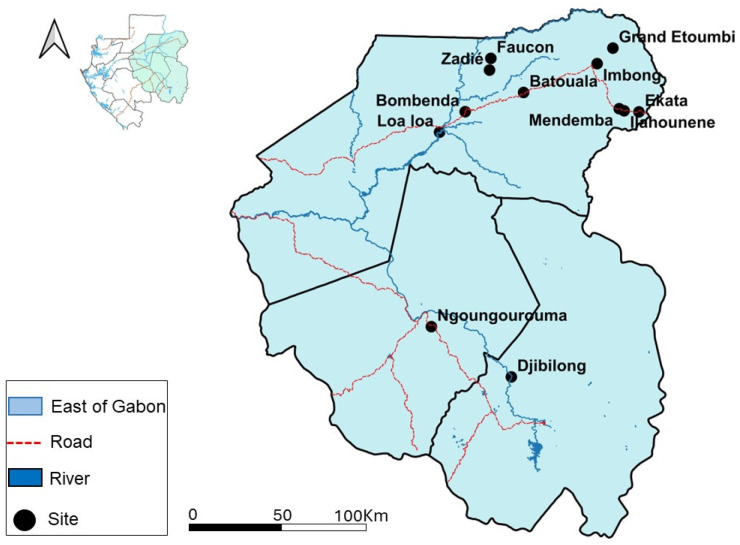
Distribution of bat collection sites in eastern Gabon. The black dots represent the collection sites.

Some bat samples were collected in the caves of Batouala (00.82 N, 13.45 E), Faucon (01.07287 N, 13.20739 E), and Zadié (00.98595 N, 13.19745 E). All three were located in the Belinga forest in the province of Ogooué-Ivindo. Djibilong Cave (−1.289466 N, 13.359246 E) was located in the Haut-Ogooué and the Ngoungourouma Cave (−0.91671 N, 12.76662 E) in Ogooué-Lolo. Other bat samples were collected around the villages Bombenda (0.67740 N, 13.01711 E), Imbong (1.03385 N, 13.9952 E), Ekata (0.67594 N, 14.305 E), Grand Etoumbi (1. 14892 N, 14.112 E), Ilahounene (0.68394 N, 14.1953 E), Mendemba (0.69959 N, 14.1587 E), and Loa loa (0.5255 N, 12.82709 E) in the Ogooué-Ivindo province (Figure 1).

Some parameters such as sex, age (juvenile, subadult, and adult), and season were recorded. Individuals found clinging to their mother’s nipples or body (approximately less than 3 months) were considered juveniles; those in whom the sexual organs (testicles for males and nipples for females) were poorly developed (at least 6 months) were considered subadults; and individuals with well-developed external morphology and sexual organs were classified as adults. All bat species were identified morphologically during the collections using the identification keys specific to African bats [24]. For some bats, morphological identification had been confirmed through genetic identification as previously described [25].

### 2.2. Sample Preparation and DNA Extraction

Due to the limited availability of materials, bat liver samples were grouped into pools containing five monospecific species at most. The liver samples were ground with 600 µL of cold Phosphate Buffer Saline (PBS) obtained from Biological Diagnostic Supplies Ltd. in a ball-mill (Geno/Grinder 2000, Spex Centripep). DNA was extracted from 200 µL of shredded supernatant using the commercial DNeasy Blood and Tissue kit (Qiagen) according to the manufacturer’s recommendations. The total number of individuals processed was 859 and was split into 180 pools.

### 2.3. BtHBV PCR Amplification and Sequencing

Bat liver DNA extracts were used for nested PCR amplification. Specific primers were used to amplify a 395 bp fragment of the surface gene (S gene) (Table S1). The S gene was used because it is a criterion for classifying HBV [26,27] based on itsgenetic variability [28,29]. The commercial Platinum Taq DNA polymerase kit produced by Invitrogen Life Technologies was used for the first and second rounds with volumes of 0.2 µL and 0.1 µL, respectively. The final volume of the two reactions was 25 µL and contained 5 µL of DNA for the first round or 1 µL of PCR product for the 2nd round, 2.5 µL of 10x buffer, 0.2 µM of dNTP, 0.02 µg of Bovine Serum Albumin (BSA), 1.5 µM of Mgcl_2_, and 0.4 µM of each of the sense and antisense primers. The positive control was a BtHBV DNA and the negative control was molecular biology water. The PCR reactions were performed using a protocol touchdown of 95 °C for 3 min, 10 cycles of 94 °C for 15 s, and 64 °C for 20 s, with a decrease of 1 °C per cycle and an elongation of 72 °C for 45 s followed by an additional 40 cycles of temperature hybridization at 54 °C as previously described [11]. PCR products were visualized after electrophoresis on 1.5% agarose gel with Gel red and under UV light using Quantum-ST4 1100/26MX software (Vilber Lourmat, 77202 Marne La Vallee, France). Samples from the positive pools were tested individually and PCR products at the expected size were Sanger-sequenced as previously described [30].

### 2.4. Phylogenetic Analysis

The obtained sequences were first assembled with ChromasPro software version 1.7.7 (Technelysium Pty, Ltd.) and then aligned with other BtHBV (surface gene) sequences from GenBank using MEGA 11. The phylogenetic tree was estimated from the 395-nucleotide alignment (accession numbers and national origins of each strain are indicated on the tree) using MEGA 11 software. The maximum likelihood method based on the Tamura–Nei model [31] was used to derive the phylogenetic analysis with 1000 bootstrap replicates. Bootstrap values are shown next to the nodes in the tree.

### 2.5. Statistical Analysis

Statistical analyses were performed using version 4.0.2 of the R software (http://cran.r-project.org, accessed on 8 November 2023). Initially, the variables in the population were compared using either Chi-squared tests (for observations greater than five) or Fisher’s exact tests (for observations less than five). Then, to evaluate the statistical significance of differences between observation pairs, the Fisher.multcomp function with Bonferroni correction from the RVAideMemoire package [32] was employed for post hoc pairwise comparisons.

To investigate how the incidence of the bat hepatitis B virus differs based on seasonality, location, and the physiological state of the bat, a mixed generalized linear binomial model was constructed using the lme4 package [33]. The response variable was the presence/absence of BtHBV in bats, while the explanatory variables were the collection environment (cave versus village), season (dry versus rainy), diet (insectivores, frugivorous, and nectarivores), sex (female and male), age (juvenile, subadult, and adult), and bat species. The city and province variables were considered as random effects. Significant interactions among the variables were estimated through the Akaike Information Criterion (AIC) for selecting models [34]. The dredge function of the MuMIn package [35] was employed to evaluate changes in AIC when model terms were added or removed. The model with the minimum AIC was deemed superior, but models with a ΔAIC < 2 were also retained [36]. Thus, the parameters for the multiple models were calculated using the MuMIn package.

## 3. Results

### 3.1. Characteristics of the Studied Population

A total of 859 bat livers belonging to 11 different species (morphologically and genetically identified), among which 444 (51.7%) were female, were analyzed. Most of the collected bats, 682 individuals (79.4%), were adults and 450/859 (52.4%) were collected in the Ogooué-Ivindo province. The most represented bat species were *Rousettus aegyptiacus* 241 (28%), *Epomops franqueti* 168 (19.6%) (frugivorous bats), *Miniopterus inflatus* 198 (23%) (insectivorous bats), and *Megaloglossus woermanni* 107 (12.5%) (nectarivorous bats). The remaining species *Coleura afra*, *Eidolon helvum*, *Hipposideros cf ruber*, *Macronycteris gigas*, *Neoromicia tenuipinnis* (insectivorous bats), and *Hypsignathus monstrosus* as well as *Myonycteris torquata* (frugivorous bats) had numbers below 45 individuals. We observed significant variability in the species and numbers of bats collected between caves and villages. Indeed, 526 (61.2%) came from caves and the rest, 333 (38.8%), were collected in the vicinity of the villages (Table 1). *Eidolon helvum*, *Epomops franqueti*, *Hypsignathus monstrosus*, *Megaloglossus woermanni*, *Myonycteris torquata*, and *Neoromicia tenuipinnis* were only collected in villages, while *Hipposideros cf ruber* and *Macronycteris gigas* were only collected in caves. The other species were collected in caves and villages (Table S2).

### 3.2. Detection of BtHBV in Bats

**Figure 2:**
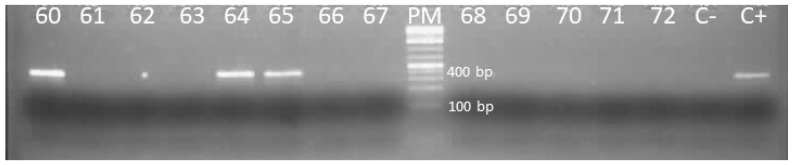
Migration of second-round PCR products obtained after amplifying a portion of the S gene. Sixty, 64, 65: positive samples; 61, 62, 63, 66, 67, 68, 69, 70, 71, 72: negative samples; PM: 100 bp molecular weight marker; C+: positive control; C-: negative control; bp: base pair.

**Figure 3:**
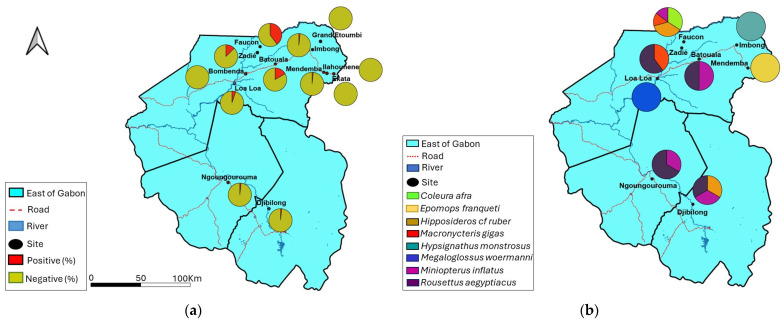
(**a**) Distribution of BtHBV PCR-positive and -negative bats according to collection sites. (**b**) Distribution of BtHBV PCR-positive bat species by collection site.

Of the 859 bat livers tested, 64 samples had DNA fragments at the expected size after nested PCR (Figure 2) giving an overall prevalence of 7.4%. Approximately 8% (55/682) of adult bats and 10.4% (43/413) of males were positive (Table 2). The number of BtHBV-positive samples in Faucon, Batouala and Zadié caves was 41 (39.8%), 2 (16.7%) and 10 (12.5%) respectively. Bombenda, Grand Etoumbi, Ekata, and Ilahounene villages had no BtHBV-positive samples (Figure 3A and Table 2). A total of eight species were positive for BtHBV. These positive species were *Coleura afra* 14 (75%), *Hipposideros cf ruber* 16 (53.3%), *Macronycteris gigas* 10 (25%), *Miniopterus inflatus* 9 (4.5%) (insectivorous bats), *Hypsignathus monstrosus* 1 (20%), *Rousettus aegyptiacus* 10 (4.1%), *Epomops franqueti* 1 (0.6%) (frugivorous bat), and *Megaloglossus woermanni* 3 (2.8%) (nectarivorous bats) (Table 2). In the Faucon cave, four species of bats were positive, namely *Coleura afra* (14/18), *Hipposideros cf ruber* (15/24), *Macronycteris gigas* (6/30), and *Miniopterus inflatus* (6/31). In the Batouala and Ngoungourouma caves, the species *Rousettus aegyptiacus* (1/6) (2/156) and *Miniopterus inflatus* (1/6) (1/4) were, respectively, found positive; in Djibilong, the species *Rousettus aegyptiacus* (1/8), *Miniopterus inflatus* (1/154), and *Hipposideros cf ruber* (1/5) were positive; and in the Zadié cave, the species *Rousettus aegyptiacus* (6/69) and *Macronycteris gigas* (4/10) were positive. Loa loa, Mendemba, and Imbong villages recorded only one BtHBV-positive species *Megaloglossus woermanni* (3/15), *Epomops franqueti* (1/27), and *Hypsignathus monstrosus* (1/1), respectively (Figure 3B and Table 2).

### 3.3. Phylogenetic Analysis

**Figure 4:**
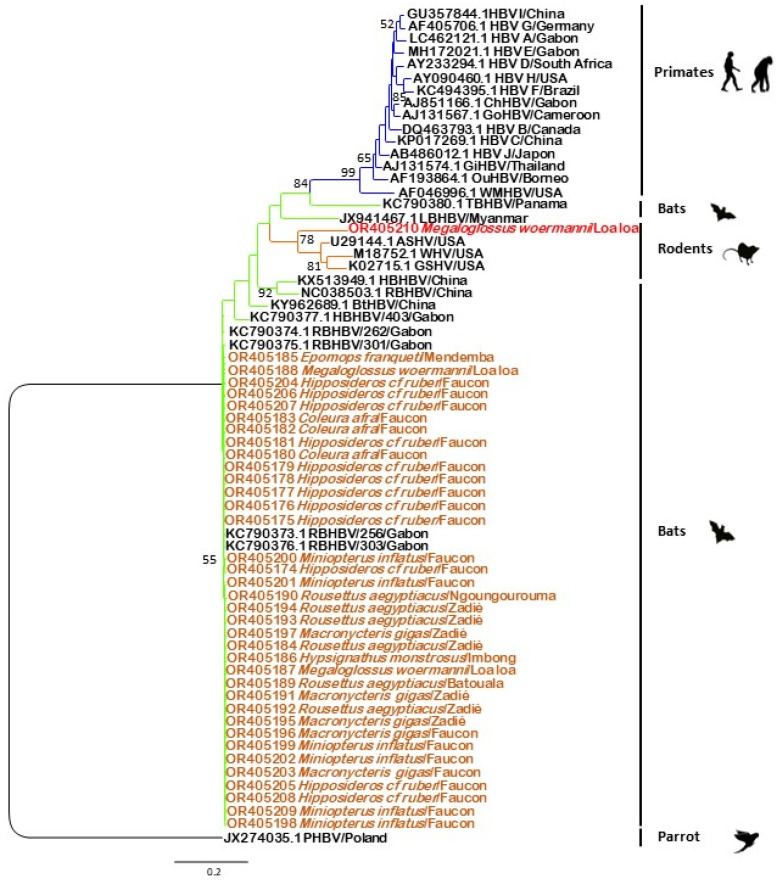
Maximum likelihood phylogenetic analysis based on the partial nucleotide sequence of the S gene. Multiple sequence alignment was performed using the ClustalW algorithm implemented in the MEGA XI software package version 11.0.13. Phylogenetic trees were constructed using the maximum likelihood method and 1000 bootstrap replicates. Only bootstrap values greater than 50% are shown on the branches. Sequences from this study are represented in red and orange, while previous ones from GenBank are represented in black.

Of the 64 PCR-positive individuals, we obtained 37 usable sequences after sequencing. The alignment was made with a portion of 395 bp of the S gene of these 37 sequences and other additional sequences available on GenBank using the NCBI BLAST “Blastn” algorithm. Phylogenetic analysis indicated that the bat sequences in this study formed two groups that belong to two different clades. The first clade included BtHBV sequences isolated in Gabon and China and the second one included HBV sequences found in rodents (Figure 4). The results showed 36 out of 37 sequences clustered with BtHBV sequences from bats isolated in Gabon and belonging to the species RBHBV, while the last sequence clustered with HBV strains found in American rodents. Among the 36 sequences, three were detected in the species *Coleura afra* (OR405180, OR405182, OR405183), 12 in the species *Hipposideros cf ruber* (OR405174 to OR405179, OR405181, OR405204 to OR405208), six in the species *Miniopterus inflatus* (OR405198 to OR405202, OR405209), and two in the species *Macronycteris gigas* (OR405196, OR405203) all coming from the Faucon cave, while the three other sequences from *Macronycteris gigas* (OR405191, OR405195, OR405197) came from the Zadié cave. The remaining sequences from *Rousettus aegyptiacus* came from Batouala (OR405189), Ngoungourouma (OR405190), and Zadié (OR405184, OR405191, OR405193 and OR405194), that from *Epomops franqueti* (OR405185) from the village Mendemba, that from *Hypsignathus monstrosus* (OR405186) from Imbong village, and that from *Megaloglossus woermanni* (OR405187, OR405188) from the village Loa loa. Whatever the location and year of collection may be, one strain is found in most cases. The 37th sequence came from *Megaloglossus woermanni* (OR405210) collected in the village Loa loa (Figure 4 and Table S5). The phylogenetic analysis based on a portion of the S gene showed 36 sequences clustered with RBHBV/GB09-256 (KC790373.1), RBHBV/GB09-303 (KC90376.1) (99.49% nucleotide similarity) and one sequence clustered with ASHV (U29144.1), WHV (M18752.1), and GSHV (K02715.1) (84.9% nucleotide similarity).

### 3.4. Factors Favoring BtHBV Positivity in Bats

Giving the disproportionality in the distribution of BtHBV-positive bats, we looked for factors explaining these differences. We made a pairwise comparison of BtHBV proportions between sex, age, species, diet, collection sites, and season to determine the parameters influencing the occurrence of infection. Comparison of the proportions of infected bats according to variables showed a statistically significant difference between sex, species (*p*-value < 0.0004998), diet, collection sites (villages vs. caves), and season. Indeed, males were more infected than females (*p*-value < 0.002432) and bats collected in caves (*p*-value < 2.608 × 10^7^) during the dry season (*p*-value < 8.497 × 10^8^) were also the most infected (Table 2). Furthermore, when comparisons were made within species, diet, and cave variables, we noted that the species *Coleura afra*, *Hipposideros cf ruber* and *Macronycteris gigas* (insectivorous bats) located in Faucon and Zadié caves were significantly impacted by BtHBV (Tables S3 and S4).

However, the generalized linear mixed model (GLMM) retained only bat species, bat lifespan, and collection site (cave or village) as significant predictors for the occurrence of BtHBV infection among bats (Table 3). Our results revealed that the probability of developing BtHBV infection in caves was significantly increased in relatively old individuals belonging to the bat species *Coleura afra* and *Hipposideros cf ruber*.

## 4. Discussion

During the last decade, many researches have focused on bats since they are considered an important reservoir of zoonotic viruses and the hosts of a genetic diversity of hepadnaviruses [37]. In this study, we analyzed 859 bat livers that were collected in three provinces (Haut-Ogooué, Ogooué-Ivindo, Ogooué-Lolo) of Gabon for the search of bat hepatitis B virus (BtHBV) homologs. The BtHBV was detected in 64 bat livers belonging to eight species among which *Coleura afra*, *Hipposideros cf ruber*, *Macronycteris gigas*, *Hypsignathus monstrosus*, *Miniopterus inflatus*, *Rousettus aegyptiacus*, *Megaloglossus woermanni*, and *Epomops franqueti* were included. A previous study conducted on 3080 samples of bats collected between 2002 and 2011 in Gabon, Australia, Brazil, Germany, Panama, and Papua New Guinea found only three positive species including *Rhinolophus alcyone*, *Hipposideros cf ruber ruber* (Gabon), and *Uroderma bilobatum* (Panama) [11]. Together with our results, these data suggest that BtHBV has been endemic in the bat community in Gabon for more than 12 years. The infection is widespread over a large part of Gabon; this tendency had already been described in Panama by Hiller and colleagues, where bats were found infected in an area of over 10,000 km^2^ [38]. In our study, we recorded a global prevalence of 7.4%. In contrast, a study by Drexler and colleagues, carried out 10 years earlier, obtained a low prevalence of 0.3% [11]. Another study conducted in Vietnam also obtained a low BtHBV prevalence of 1.3% [39]. However, studies conducted in China in 2017 and 2019 recorded BtHBV prevalences of 13.3% and 6.6%, respectively [40,41]. BtHBV circulates in Asian, African, and American bats at different frequencies. The sample size and collection sites could explain the difference in prevalences observed.

In addition to the 19 bat species previously found positive for BtHBV (*Rhinolophus alcyone*, *Rhinolophus ferrumequinum*, *Rhinolophus luctus*, *Rhinolophus monoceros*, *Rhinolophus pearsonii*, *Rhinolophus pusillus*, *Rhinolophus sinicus*, *Hipposideros* cf. *ruber*, *Hipposideros larvatus*, *Hipposideros Pomona*, *Hipposideros armiger*, *Miniopterus fuliginosus*, *Miniopterus schreibersii*, *Myotis chinensis*, *Myotis davidi*, *Myotis fimbriatus*, *Myotis pequinius*, *Myotis ricketti,* and *Uroderma bilobatum*) [10,11,40,41,42,43,44], we detected BtHBV in seven (7) additional species for the first time in this study which are as follows: *Coleura afra*, *Macronycteris gigas*, *Hypsignathus monstrosus*, *Miniopterus inflatus*, *Rousettus aegyptiacus*, *Megaloglossus woermanni*, and *Epomops franqueti*. These new data suggest that BtHBV is gradually infecting new bat species. However, the previous presence of BtHBV in these bat species could not be excluded as few longitudinal studies have been conducted. One of these genera, *Hipposideros*, has been found positive in three countries (China [40,41,42,43], Gabon [11], and Myanmar [10]) suggesting that this bat genus may be the natural reservoir for BtHBV. Taken together, these data suggest that bats represent an important reservoir of *Orthohepadnavirus*. The BtHBV-negative bat species in our study could be due to the small sample size. Additional sampling would be necessary to confirm or invalidate the present results.

The highest BtHBV prevalences were found in the Zadié (12.5%), Batouala (16.7%), and Faucon (39.8%) caves where most bats are insectivorous. In contrast, the prevalence observed around villages, where most bats are frugivorous and nectarivorous and only five bats were found positive, was low at 1.5%. In 2018, Ling’en Yang and colleagues recorded a similar result with a BtHBV prevalence of 1.6% in bats collected near humans [44]. Meanwhile, in 2017, Fang-Yuan Nie and colleagues and in 2019, Si-Cong Lei and ccolleagues, recorded BtHBV prevalences in bats from caves of 13% and 6.6%, respectively [40,41]. The difference in BtHBV prevalence between caves and villages could be explained by the ease with which bats move between caves and their diets. The caves of Batouala and Zadié are 33.7 km apart, those of Zadié and Faucon are 11.7 km apart, and those of Batouala and Faucon are 37.7 km apart [45]. By changing their roosting and feeding locations based on food availability, bats adopt a migratory behavior responsible for spreading pathogens over considerable distances as documented for fruit bats capable of migrating over 1500 km [46]. Studies have also shown that feeding niches and feeding behaviors can contribute to a bat’s exposure to and spread of viral pathogens [47]. The results obtained during this study suggest that caves are a critical site for BtHBV carriage in bats. Many studies of bats have rejected the hypothesis that caves promote viral richness. However, collected data are consistent with the hypothesis that cave bats have a higher probability of viral sharing, especially those roosting in the same cave [47]. Caves are favorable shelters for the rearing of the young and offer protection against bad weather and predators.

Therefore, some bat species can share a square meter with more than 450 individuals and more than 10 bat species can share the same cave with about 20 million individuals [47]. In addition, the viral sharing of BtHBV observed in different bat species could be explained by their resting habitats and the increasing pressure on cave ecosystems due to human activity. In some parts of Africa, caves are used as hunting grounds for bats that are consumed as game as well as for the exploitation of minerals [47,48,49]. In Gabon, the Zadié, Faucon, and Batouala caves are subject to intense human activity due to the hunting activities there. This anthropic stress could increase viral excretion in the bat population and increase the frequency of exposure and contact between infected and healthy bats. However, it also poses a threat to humans [47]. Thus, the proximity of humans to bats and the existence of a strain of BtHBV capable of infecting human hepatocytes, as demonstrated by Drexler and colleagues [11], constitutes a health risk for these populations.

To date, there are four species of BtHBV in bats classified within the genus of *Orthohepadnavirus*. These are the tent-making bat hepatitis B virus (TBHBV) [10], long-fingered bat hepatitis B virus (LBHBV), horseshoe bat hepatitis B virus (HBHBV), and roundleaf bat hepatitis B virus (RBHBV) [11]. Phylogenetic analysis indicated that 36 sequences (97.3%) of the bat hepadnaviruses found in this study were closely related to the RBHBV previously detected in Gabon, suggesting that RBHBV is the most common BtHBV species found in Gabonese bats. The RBHBV sequences display up to 100% nucleotide identity, confirming that there are few barriers to prevent interspecific transmission of hepadnaviruses in bats as demonstrated by Fang-Yuan Nie et al. [40].

On the other hand, one sequence detected in *Megaloglossus woermanni* was closely related to HBV strains found in rodents in America [5,6,7] with 84.9% nucleotide identity, suggesting a circulation and cross-species transmission of hepadnaviruses between rodents and bats, particularly in *Megaloglossus woermanni*. Together with the fact that bats are carriers of a diversity of hepadnaviruses, such as TBHBV, that have the capacity to infect primary human hepatocytes [10,11,44], these results support the hypothesis that bats may be ideal hosts for ancestral hepadnaviruses [11,44] and suggest that some hepadnaviruses may be specific to one bat species. In our study, we amplified a portion of the S gene. However, HBV classification must be based on either the S gene or the complete genome, with a nucleotide divergence of at least 4% and 7.5%, respectively, to define a new genotype [26,27]. Additional analyses are therefore necessary to determine whether we are in the presence of a new genotype.

We also found that two bats from the species *Megaloglossus woermanni* collected in the same village were carriers of the RBHBV strain found in other species, such as *Epomops franqueti* and *Rousettus aegyptiacus*, that have different diets. This result could be explained through the migratory movements of certain bats looking for new food niches or even a change in habitat due to deforestation. Jesus Olivero and colleagues demonstrated that human activities can increase the geographical range of these bats, thus influencing the spread and transmission of certain viruses, as has been demonstrated in the case of the Ebola virus [50].

Paired analysis of parameters that could influence the occurrence of BtHBV infection in bats revealed a significant difference between males and females, bat species, diet, habitat, and season. A study by Thomas Hiller and colleagues highlighted the anthropogenic factors affecting hepadnavirus infection in a neotropical bat. Among these factors were geographical location, female sex, and no breeding status [38]. However, in our study, most BtHBV-positive individuals were adults, nine were subadults, and none were juveniles. This does not allow us to conclude that age parameter could influence the infection. The determinants that might favor viral sharing are yet to be well known. Nevertheless, some studies have examined some bat species’ ecological traits and life histories associated with viral richness. These include geographic range, sympatry, the genetic structure of the population, colony size, body mass, and alimentation [47]. In our study, only some of these parameters were available. Additional longitudinal studies are therefore needed to support the results of this study.

A GLMM analysis was conducted to gain insights into the determinants influencing the occurrence of BtHBV in bats. The results identified the variable species as the most effective model for explaining BtHBV occurrence. Out of the collected species, *Coleura afra* and *Hipposideros cf ruber* (insectivorous bats) were found to be the most susceptible to infection. Previous studies have shown that these two bat species cohabit and have a similar diet [51]. In addition, the pairwise analysis undertaken during this study has shown that insectivorous bats are more infected than others. This observation reinforces that niche feeding and feeding behaviors are associated with the exposure and transmission of viral pathogens in bats [47]. These bats would have specific proteins on their Sodium Taurocholate Co-transporting Polypeptide (NTCP) receptors which BtHBV uses to infect hepatocytes. The study of the evolution of this receptor in primates, rodents, and bats revealed differences in the genetic fingerprints of NTCP, making it a limiting factor in BtHBV infectivity [52]. However, in bats, the NTCP appears to be a weak genetic constraint, thus increasing the transmission of orthohepadnaviruses between different species. On the other hand, researchers have hypothesized that BtHBV uses additional genetic fingerprints to infect hepatocytes [52]. This could explain the differences in the prevalence infection observed between different species in this study.

## 5. Conclusions

In conclusion, this study reveals a significant prevalence of hepadnaviruses among Gabonese bats. Some bat species previously identified as carriers of hepadnaviruses were tested positive in this study, while others were newly identified as BtHBV hosts. Our findings provide evidence of high interspecies transmission and a low genetic barrier for these viruses in bats. The BtHBV sequence homologous to those found in rodents indicate that bats might be a significant reservoir of ancestral hepadnaviruses. Our findings, and those of other researchers, suggest that numerous BtHBV species are likely to circulate in many different bat species, presenting a potential zoonotic danger and a public health risk. Large-scale and longitudinal research across various bat families is necessary to evaluate the zoonotic potential of these viruses concerning humans.

**Limits:** We tried to obtain the complete genomes of viruses identified during this study by selecting 10 samples among the 37 from which we obtained Sanger sequences. However, we could not obtain any amplification. This could be due not only to the low BtHBV viral load but also due to high levels of impurities and the host genome in liver samples that render the NGS method less efficient. Indeed, previous studies have shown that bats can maintain relatively low levels of viral load [53] and PCR amplification followed by sequencing on samples with a low viral load and/or high genetic variability gives better results in obtaining a complete HBV genome than direct NGS [54,55]. Additional studies on a larger sample of bats are needed to conclude the determinants that might favor BtHBV occurrence and to obtain the complete genome sequences of the BtHBV detected during this study.

## Figures and Tables

**Table 1:** Characteristics of the study population.

Variables	Number of Samples n = 859 (%)	Haut-Ogooué n = 167 (%)	Ogooue-Ivindo n = 528 (%)	Ogooué-Lolo n = 164 (%)
**Age**				
J	26 (3)	5 (3)	15 (2.8)	6 (3.7)
SA	149 (17.4)	12 (7.2)	62 (11.8)	75 (45.7)
A	**682 (79.4)**	**149 (89.2)**	**450 (85.2)**	**83 (50.6)**
Unknown	2 (0.2)	1 (0.6)	1 (0.2)	-
**Sex**				
Males	413 (48.1)	**112 (67.1)**	238 (45.1)	63 (38.4)
Females	**444 (51.7)**	55 (32.9)	289 (54.7)	100 (61)
Unknown	2 (0.2)	-	1 (0.2)	1 (0.6)
**Species**				
*Coleura afra*	18 (2.1)	-	18 (3.4)	-
*Eidolon helvum*	2 (0.2)	-	2 (0.4)	-
*Epomops franqueti*	168 (19.6)	-	**168 (31.9)**	-
*Hipposideros cf ruber*	30 (3.5)	5 (3)	25 (4.7)	-
*Macronycteris* *gigas*	44 (5.1)	-	40 (7.6)	4 (2.4)
*Hypsignathus monstrosus*	5 (0.6)	-	5 (0.9)	-
*Megaloglossus woermanni*	107 (12.5)	-	107 (20.3)	-
*Miniopterus inflatus*	198 (23)	154 (92.2)	40 (7.6)	4 (2.4)
*Myonycteris torquata*	10 (1.2)	-	10 (1.9)	-
*Neoromicia tenuipinnis*	5 (0.6)	-	5 (0.9)	-
*Rousettus aegyptiacus*	**241 (28)**	8 (4.8)	77 (14.6)	**156 (95.2)**
Unidentified	31 (3.6)	-	31 (5.8)	-
**Diet**				
Insectivorous	295 (34.3)	159 (95.2)	128 (24.2)	8 (4.9)
Frugivorous	**426 (49.6)**	8 (4.8)	262 (49.6)	156 (95.1)
Nectarivorous	107 (12.5)	-	107 (20.3)	-
Unidentified	31 (3.6)	-	31 (5.9)	-
**Collection sites**				
**Caves**				
Djibilong	**167 (31.7)**	**167 (100)**	-	-
Batouala	12 (2.3)	-	12 (6.2)	-
Faucon	103 (19.6)	-	**103 (52.8)**	-
Zadié	80 (15.2)	-	80 (41)	-
Ngoungourouma	164 (31.2)	-	-	**164 (100)**
Total	526 (100)	167	**195**	164
**Villages**				
Bombenda	10 (3)	-	10 (3)	-
Ekata	34 (10.2)	-	34 (10.2)	-
Grand Etoumbi	48 (14.4)	-	48 14.4)	-
Ilahounene	**68 (20.4)**	-	**68 (20.4)**	-
Imbong	55 (16.5)	-	55 (16.5)	-
Loa loa	58 (17.4)	-	58 (17.4)	-
Mendemba	60 (18.1)	-	60 (18.1)	-
Total	333 (100)	-	333	-
**Season**				
Dry season	**460 (53.6)**	-	**460 (87.1)**	-
Rainy season	399 (46.4)	**167 (100)**	68 (12.9)	**164 (100)**

J: juvenile; SA: subadult; A: adult.

**Table 2:** BtHBV PCR-positive bats.

		Positive PCR (%)				
Variables	Number of Samples n = 859(%)	Haut-Ogooué n = 167(%)	Ogooue-Ivindo n = 528(%)	Ogooué-Lolo n = 164(%)	Total Positive PCR (%)	Fisher Test/Chi Test (*p*-Value)
**Age**						
J	26	0/5	0/15	0/6	0/26	
SA	149	0/12	8/62 (12.9)	1/75 (1.3)	9/149 (6)	
A	682	3/149 (2)	50/450 (11.1)	2/83 (2.4)	**55/682 (8)**	0.397
Unknown	2	0/1	0/1	-	0/2 (0)	
**Sex**						
Males	413	1/112 (0.9)	39/238 (16.4)	3/63 (4.8)	**43/413 (10.4)**	
Females	444	2/55 (3.6)	19/289 (6.6)	0/100	21/444 (4.7)	0.002432 **
Unknown	2	-	0/1	0/1	0/2	
Species						
*Coleura afra*	18	-	14/18 (75)	-	**14/18 (75)**	
*Eidolon helvum*	2	-	0/2	-	0/2	
*Epomops franqueti*	168	-	1/168 (0.6)	-	1/168 (0.6)	
*Hipposideros cf ruber*	30	1/5 (20)	15/25 (60)	-	**16/30 (53.3)**	
*Macronycteris* *gigas*	44	-	10/40 (25)	0/4	10/44 (25)	
*Hypsignathus monstrosus*	5	-	1/5 (20)	-	1/5 (20)	
*Megaloglossus woermanni*	107	-	3/107 (2.8)	-	3/107 (2.8)	0.0004998 ***
*Miniopterus inflatus*	198	1/154 (0.6)	7/40 (17.5)	1/4 (25)	9/198 (4.5)	
*Myonycteris torquata*	10	-	0/10	-	0/10	
*Neoromicia tenuipinnis*	5	-	0/5	-	0/5	
*Rousettus aegyptiacus*	241	1/8 (12.5)	7/77 (9.1)	2/156 (1.3)	10/241 (4.1)	
Unidentified	31	-	0/31	-	0/31	
**Diet**						
Insectivorous	295	2/159 (1.3)	46/128 (35.9)	1/8 (12.5)	**49/295 (16.6)**	
Frugivorous	426	1/8 (12.5)	9/262 (0.8)	2/156 (1.3)	12/426 (2.8)	4.312 × 10^11^ ***
Nectarivorous	107	-	3/107 (2.8)	-	3/107 (2.8)	
Unidentified	31	-	0/31	-	0/31	
**Collection sites**						
**Caves**						
Djibilong	167	3/167 (1.8)	-	-	3/167 (1.8)	
Batouala	12	-	2/12 (16.7)	-	2/12 (16.7)	
Faucon	103	-	41/103 (39.8)	-	**41/103 (39.8)**	
Zadié	80	-	10/80 (12.5)	-	10/80 (12.5)	
Ngoungourouma	164	-	-	3/164 (1.8)	3/164 (1.8)	
**Villages**						2.608 × 10^7^ ***
Bombenda	10	-	0/10	-	0/10	
Ekata	34	-	0/34	-	0/34	
Grand Etoumbi	48	-	0/48	-	0/48	
Ilahounene	68	-	0/68	-	0/68	
Imbong	55	-	1/55 (1.8)	-	1/55 (1.8)	
Loa loa	58		3/58 (5.2)	-	3/58 (5.2)	
Mendemba	60	-	1/60 (1.7)	-	1/60 (1.7)	
**Season**						
Dry season	460	-	55/460 (12)	-	**55/460 (12)**	8.497 × 10^8^ ***
Rainy season	399	3/167 (1.8)	3/68 (4.4)	3/164 (1.8)	9/399 (2.3)	

J: juvenile; SA: subadult; A: adult; ** *p* < 0.01; *** *p* < 0.001.

**Table 3:** Result of the generalized linear mixed model to explain the occurrence of hepatitis B virus in bats.

Model No. (Rank)	Fixed Effects						Df	ΔAIC	Akaike Weight
	Intercept	Age	Location	Season	Sex	Species			
**17 (1)**	**−3.747**					+	14	0	0.216
**18 (2)**	−3.513	+				+	17	0.28	0.187
**19 (3)**	−3.667		+			+	15	1.70	0.092

+ designates the factors favoring the occurrence of BtHBV in bats.

## Data Availability

Request for data should be addressed to DSKM.

## Supplementary Materials

The following supporting information can be downloaded at: https://www.mdpi.com/article/10.3390/v16071015/s1, Table S1: Oligonucleotides used for PCR screening; Table S2: Number of bats by collection sites; Table S3: Comparison of BtHBV occurrence between different caves; Table S4: Comparison of BtHBV occurrence between different species; Table S5: BtHBV bat positive species according to the year of collection.
