# Supplementary material for: Prevalence and Genetic Diversity of Bat Hepatitis B Viruses in Bat Species Living in Gabon
Source: Viruses. 2024 Jun 25;16(7):1015. doi: 10.3390/v16071015 (PMC11281422; doi:10.3390/v16071015)
Supplement: Supplementary file 1 [file viruses-16-01015-s001.zip › Table S1.pdf]

Table S1. Oligonucleotides used for PCR screening

Target region	Primer names	Sequence (5′–3′)	Polarity	Round	Sizes (pb)	Reference
Surface	HBVall-F1364	CTAGATTSGTGGTGGAYTTCTCTC	+	First	413	(Drexler et al., 2013)
	HBVall-R1620a	GAGAAAMGGRCTGAGRCCSACTCCCAT	-			
	HBVall-R1620b	GAGAAAMGGRGAGAGRCCSACTCCCAT	-			
	HBVall-F1372	GTGGTGGAYTTCTCTCAGTTYTC	+	Second	395	
	HBVall-R1610a	CTGAGRCCSACTCCCATWGG	-			
	HBVall-R1610b	GAGAGRCCSACTCCCATWGG	-			
