# Supplementary material for: Prevalence and Genetic Diversity of Bat Hepatitis B Viruses in Bat Species Living in Gabon
Source: Viruses. 2024 Jun 25;16(7):1015. doi: 10.3390/v16071015 (PMC11281422; doi:10.3390/v16071015)
Supplement: Supplementary file 1 [file viruses-16-01015-s001.zip › Table S2.pdf]

**Table S2.** Number of bats by collection sites

| | Bat species | | | | | | | | | | | |
|---|---|---|---|---|---|---|---|---|---|---|---|---|
| | Ca | Eh | Ef | H r | Hg | Hm | Mw | Mi | Mt | Nt | Ra | U |
| **Caves** | | | | | | | | | | | | |
| Batouala | - | - | - | - | - | - | - | 6 | - | - | 6 | - |
| Faucon | 18 | - | - | 24 | 30 | - | - | 31 | - | - | - | - |
| Zadié | - | - | - | 1 | 10 | - | - | - | - | - | 69 | - |
| Djibilong | - | - | - | 5 | - | - | - | 154 | - | - | 8 | - |
| Ngoungourouma | - | - | - | - | 4 | - | - | 4 | - | - | 156 | - |
| **Villages** | | | | | | | | | | | | |
| Bombenda | - | - | 3 | - | - | - | 4 | - | - | - | - | 3 |
| Ekata | - | - | 18 | - | - | 1 | 10 | 1 | 3 | - | - | 1 |
| Grand Etoumbi | - | - | 23 | - | - | 1 | 19 | 1 | 2 | - | - | 2 |
| Ilahounene | - | - | 48 | - | - | 1 | 11 | 1 | 1 | 4 | - | 2 |
| Imbong | - | 1 | 28 | - | - | 1 | 20 | - | 3 | - | 2 | - |
| Loa loa | - | - | 21 | - | - | - | 15 | - | - | - | - | 22 |
| Mendemba | - | 1 | 27 | - | - | 1 | 28 | - | 1 | 1 | - | 1 |

Ca *Coleura afra* ; Eh *Eidolon helvum* ; Ef *Epomops franqueti* ; Hr *Hipposideros cf ruber* ; Mg *Macronycteris gigas* ; Hm *Hypsignathus monstrosus* ; Mw *Megaloglossus woermanni* ; Mi *Miniopterus inflatus* ; Mt *Myonycteris torquata ;* Nt *Neoromicia tenuipinnis* ; Ra *Rousettus aegyptiacus* ; U Unidentified
