# Supplementary material for: Prevalence and Genetic Diversity of Bat Hepatitis B Viruses in Bat Species Living in Gabon
Source: Viruses. 2024 Jun 25;16(7):1015. doi: 10.3390/v16071015 (PMC11281422; doi:10.3390/v16071015)
Supplement: Supplementary file 1 [file viruses-16-01015-s001.zip › Table S3.pdf]

Table S3. Comparison of BtHBV occurrence between different caves

**Caves**	Djibilong	Batouala	Faucon	Zadié
Batouala	0.3692	-	-	-
Faucon	**8.3663e-16*** **	1	-	-
Zadié	9.511e-03**	1	5.163e-04***	-
Ngoungourouma	1	0.3812	**1.354e-15*** **	0.01*

*p<0.05; **p<0.01 ; ***p<0.001
