# Supplementary material for: Prevalence and Genetic Diversity of Bat Hepatitis B Viruses in Bat Species Living in Gabon
Source: Viruses. 2024 Jun 25;16(7):1015. doi: 10.3390/v16071015 (PMC11281422; doi:10.3390/v16071015)
Supplement: Supplementary file 1 [file viruses-16-01015-s001.zip › Table S4.pdf]

Table S4. Comparison of BtHBV occurrence between different species

**Species**	*Coleura afra*	*Eidolon Elvum*	*Epomops franqueti*	*Hipposideros cf ruber*	*Macronycteris gigas*	*Hypsignathus monstrosus*	*Megaloglossus woermanni*	*Miniopterus inflatus*	*Myonicteris torquata*	*Neoromicia tenuipinnis*	*Rousettus aegyptiacus*
Eidolon Elvum	1	-	-	-	-	-	-	-	-	-	-
Epomops franqueti	**7.2e-15*****	1	-	-	-	-	-	-	-	-	-
Hipposideros cf ruber	1	1	**1.06e-12*****	-	-	-	-	-	-	-	-
Macronycteris gigas	**6.786e-03*****	1	**3.74e-05*****	**0.82**	-	-	-	-	-	-	-
Hypsignathus monstrosus	1	1	1	1	1	-	-	-	-	-	-
Megaloglossus woermanni	**1.01e-10*****	1	1	**2.22e-08*****	**0.02***	1	-	-	-	-	-
Miniopterus inflatus	**3.87e-11*****	1	1	**7.32e-09*****	**0.03***	1	1	-	-	-	-
Myonycteris torquata	**0.01***	1	1	**0.17**	1	1	1	1	-	-	-
Neoromicia tenuipinnis	**0.247**	1	1	1	1	1	1	1	1	-	-
Rousettus aegyptiacus	**6.901e-12*****	1	1	**1.15e-9*****	**0.01**	1	1	1	1	1	-

*p<0.05 ; **p<0.01 ; ***p<0.001
