# Supplementary material for: Prevalence and Genetic Diversity of Bat Hepatitis B Viruses in Bat Species Living in Gabon
Source: Viruses. 2024 Jun 25;16(7):1015. doi: 10.3390/v16071015 (PMC11281422; doi:10.3390/v16071015)
Supplement: Supplementary file 1 [file viruses-16-01015-s001.zip › Table S5.pdf]

Table S5. BtHBV bat positive species according to the year of collection

			**Positive PCR (%)**			
			**Number of samples collected per year**			
**Variables**		**Number of samples** N=859(%)	**2013** n=362(%)	**2014** n=164(%)	**2017** n=68(%)	**2019** n=265(%)
**Haut-Ogooué**	**Species**					
	Hipposideros cf ruber	5	1/5 (20)	-	-	-
	Miniopterus inflatus	154	1/154 (0.65)	-	-	-
	Rousettus aegyptiacus	8	1/8 (12.5)	-	-	-
**Ogooué-Ivindo**						
	Coleura afra	18	**14/18 (77.8)**	-	-	-
	Eidolon helvum	2	-	-	-	0/2
	Epomops franqueti	168	-	-	0/24	1/144 (0.7)
	Hipposideros cf ruber	25	**15/25 (60)**	-	-	-
	Macronycteris gigas	40	10/40 (25)	-	-	-
	Hypsignathus monstrosus	5	-	-	-	1/5 (20)
	Megaloglossus woermanni	107	-	-	3/19 (15.8)	0/88
	Miniopterus inflatus	40	7/37 (18.9)	-	-	0/3
	Myonycteris torquata	10	-	-	-	0/10
	Neoromicia tenuipinnis	5	-	-	-	0/5
	Rousettus aegyptiacus	77	7/75 (9.3)	-	-	0/2
	Unidentified	31	-	-	0/25	0/6
**Ogooué-Lolo**						
	Macronycteris gigas	4	-	0/4	-	-
	Miniopterus inflatus	4	-	1/4 (25)	-	-
	Rousettus aegyptiacus	156	-	2/156 (1.3)	-	-
